# Fatty acid synthase reprograms the epigenome in uterine leiomyosarcomas

**DOI:** 10.1371/journal.pone.0179692

**Published:** 2017-06-27

**Authors:** Min Guan, Xiwei Wu, Peiguo Chu, Warren A. Chow

**Affiliations:** 1 Department of Medical Oncology and Therapeutics Research, City of Hope, Duarte, CA, United States of America; 2 Department of Molecular Medicine, Beckman Research Institute of the City of Hope, Duarte, CA, United States of America; 3 Department of Pathology, City of Hope, Duarte, CA, United States of America; Texas A&M University, UNITED STATES

## Abstract

SK-UT-1 uterine leiomyosarcomas (Ut-LMS) cells were transduced with a fatty acid synthase (FASN)-containing retroviral vector to recapitulate the “lipogenic phenotype of cancer.” Consistent with this model, forced expression of FASN enhanced SK-UT-1 proliferation, migration, and cellular motion. Further investigation showed FASN promotes trimethylation of H3K9 (H3K9me3) and acetylation of H3K27 (H3K27ac) in SK-UT-1 cells. In contrast, siRNA targeting of FASN in high endogenous FASN expressing SK-LMS-1 Ut-LMS cells inhibits trimethylation of H3K9 and acetylation of H3K27. Palmitate, the predominant fatty acid product of FASN, increased H3K9me3, H3K27ac and H3K27me3 detection in SK-UT-1 cells. FASN promoted histone 3 methylation and acetylation through alteration of histone 3-modifying enzymatic activities (HDAC, HDM, HMT and HAT). ChIP-seq in SK-UT-1-FASN cells with anti-H3K9me3 antibody identified regions of enriched binding compared to vector-only cells. One differentially-enriched gene, *CRISP1*, was investigated further by ChIP-PCR. The transcriptionally repressive function of H3K9me3 was confirmed in *CRISP1*. Our results provide mechanistic insight into the pathobiology of the “lipogenic phenotype of cancer.” Here, FASN reprograms the Ut-LMS epigenome through chromatin remodeling to promote the “malignant phenotype.”

## Introduction

Soft-tissue sarcomas (STS) are uncommon tumors of mesenchymal origin that consist of 70 subtypes [1]. Uterine leiomyosarcomas (Ut-LMS) are rare, highly malignant tumors arising from the smooth muscle of the uterus; they account for only 3% of all Ut cancers [2]. Ut-LMS metastasize early due to hematogenous spread. The 5-year overall survival after hysterectomy for localized Ut-LMS is only ~35% [2]. Ut-LMS recurrence is rarely curable despite aggressive surgery and chemotherapy.

The “lipogenic phenotype” of cancer occurs as lipogenic enzymes, including fatty acid synthase (FASN), are upregulated during oncogenesis-mediated metabolic reprogramming [3, 4]. FASN catalyzes *de novo* long-chain fatty acid (FA) synthesis from acetyl–coenzyme A (acetyl-CoA), malonyl-CoA and NADPH. Another rate-limiting lipogenic enzyme is acetyl-CoA carboxylase (ACC), which catalyzes the ATP-dependent carboxylation of acetyl-CoA to form malonyl-CoA [5]. The FAs are used by cancer cells as an energy source distinct from glucose and amino acids for: membrane synthesis, energy production, and post-translational protein modification. Further, FASN and *de novo* FA biosynthesis may regulate other cancer-related signaling networks.

Lipogenic enzymes including FASN are upregulated in epithelial cancers, and correlate with a higher risk of both disease recurrence and death [3, 4]. Similarly, FASN is a prognostic biomarker in STS for both reduced disease-free (DFS) and overall survival (OS) [6]. The regulation of FASN involves transcriptional and post-transcriptional control [7]. Akt/mTORC1 signaling is frequently activated in cancer, and leads to upregulation of sterol regulatory element-binding protein-1c (SREBP-1c), which transcriptionally activates FASN [8, 9]. Other mechanisms of FASN overexpression include gene copy gain and deubiquitination [10, 11].

**Fig 1:**
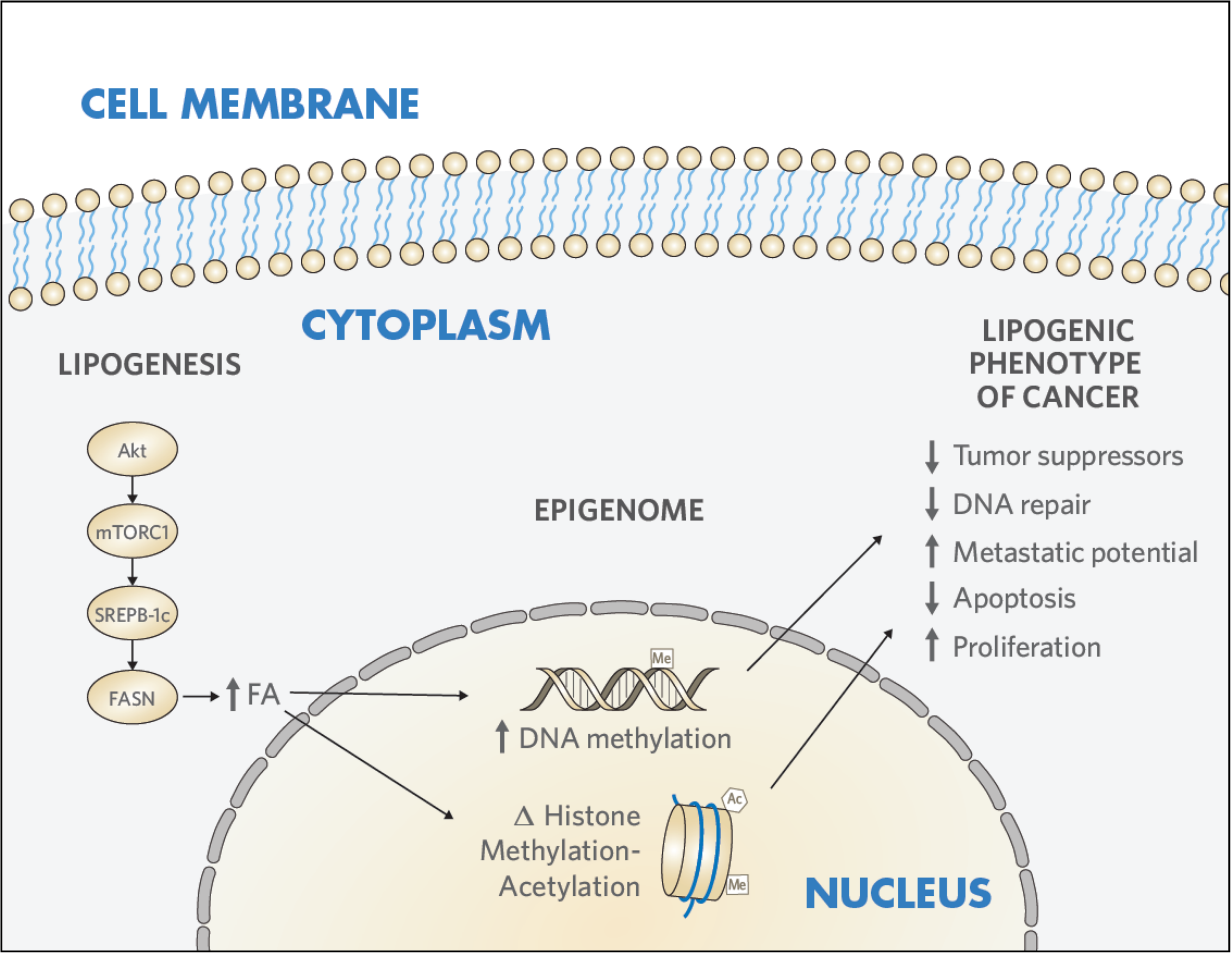
The lipogenic phenotype reprograms the epigenome in Ut-LMS. Enhanced lipogenesis modifies the epigenome to transform mesenchymal cells in Ut-LMS. **Akt**, protein kinase B; **mTORC1**, mammalian target of rapamycin complex 1; **FA**, fatty acid; **FASN**, fatty acid synthase; **SREBP-1c**, sterol regulatory element-binding protein-1c; **Ut-LMS**, uterine leiomyosarcoma.

In the current study, we developed a model of the “lipogenic phenotype” in Ut-LMS through FASN overexpression, which generated features characteristic of the malignant phenotype. We further explored the interrelationship between enhanced lipogenesis and epigenetic reprogramming in Ut-LMS. We found that FASN induced histone 3 (H3) remodeling by altering H3-modifying enzymatic activities. These results demonstrate that FASN reprograms the Ut-LMS epigenome through mediating histone modification signatures and chromatin remodeling to promote the “malignant phenotype” (Fig 1).

## Materials and methods

### Cell culture

Human Ut-LMS SK-UT-1 and SK-LMS-1 cell lines were purchased from the American Type Culture Collection (Manassas, Virginia), and were maintained in Eagle’s Minimum Essential Medium (MEM; Life Technologies, INC., Logan, UT) with 10% fetal bovine serum. FASN-expressing (-FASN) or empty vector (-EV) SK-UT-1 cell lines were produced by transduction with retrovirus prepared from pBabe-FASN-HA-Flag and pBabe-EV-HA-Flag plasmids (Dr. Massimo Loda, Dana-Farber Cancer Institute, Boston, MA).

Non-transformed “conditionally reprogrammed cells” (CRCs) have been established from patient-derived Ut-LMS in collaboration with Dr. Xuefeng Liu (Georgetown University, Washington, DC). CRC-2 cells were maintained in complete F medium: 3:1 of DMEM: F12 medium with hydrocortisone, EGF, insulin, cholera toxin and ROCK inhibitor Y-27632 as described [12]. Our protocol to develop sarcoma conditionally reprogrammed cell (CRC) lines was approved by the City of Hope Institutional Review Board. The IRB # is 15243, and written informed consent was obtained from the patient with Ut-LMS for the derivation and use of CRC.

### Chemicals and reagents

Palmitic acid was purchased from Sigma-Aldrich (St. Louis, MO). FlexiTube GeneSolution GS2194 for FASN and All Stars control siRNA were purchased from Qiagen (Hilden, Germany), and used for transfection with RNAiMAX transfection reagent (Invitrogen, Carlsbad, CA). FASN antibodies were purchased from Santa Cruz Biotechnology, Inc. (Santa Cruz, CA) and H3K4me3, H3K9me3, H3K27me3 and H3K27ac antibodies was were purchased from Abcam, Inc. (Cambridge, MA). Human fibronectin was purchased from Corning, Inc (Corning, NY).

### Western blot analysis

Stable FASN-expressing (-FASN) or empty vector (-EV) SK-UT-1 cell lines were selected and cultured for Western blot analysis. Parental SK-UT-1 cells were treated with 10, 100, 1000 μM palmitate for 24 hr, and cell lysates were collected to detect expression of histone 3 methylated and acetylated lysines by Western blot. FASN or scramble control siRNA were transfected into SK-LMS-1 cells and lysates were collected at the indicated time points to detect FASN and histone 3 expression.

### Histone 3-modifying enzymes activities

Histone-modifying enzymatic activity assay kits including histone deacetylase (HDAC), demethylase (HDM), acetyl transferase (HAT) and methyl transferase (H3K9 HMT) were purchased from Epigentek (Farmingdale, NY). Nuclear lysates from SK-UT-1-EV and -FASN cells were extracted and analyzed by ELISA according to the manufacturer’s protocols.

### ChIP sequencing and data analysis

Chromatin immunoprecipitation (ChIP) were performed on SK-UT-1-FASN and SK-UT-1-EV cells using the Magna ChIP A/G kit from (Millipore, Temecula, CA) following the manufacturer’s protocol. Anti-H3K9me3 antibody was used to immunoprecipitate cross-linked and fragmented chromatin DNA. This was followed by elution of protein/DNA complexes, reversal of the cross-links, and DNA purification. Library preparation and massively parallel DNA sequencing (ChIP-seq) was performed with eluted DNA using an Illumina Hiseq 2500 sequencer by the City of Hope Integrative Genomics Core (Duarte, CA). Reads were aligned to hg19 genome assembly using Novoalign and only reads aligned to a unique genome location were reported. The aligned reads were then subject to peak calling using custom R scripts and Bioconductor package “chipseq”. Reads were extended to 200bp and regions that are longer than 100bp, with at least 10 reads in the enriched sample, and with 2.5-fold enrichment compared to the input sample, were considered as peaks. Peaks were annotated by overlapping to RefSeq mRNA database, and visualized by Integrated Genomics Viewer (IGV). The reads falling into these peaks in each sample were counted and differential peaks with 2-fold or more enrichment differences between the two cell lines were identified.

### Quantitative ChIP-PCR and RT-PCR

Chromatin immunoprecipitation quantitative PCR (ChIP-PCR) was conducted using above the ChIP-eluted DNA as template. Quantitative reverse transcriptional PCR (q-RT-PCR) was carried out in total RNA extracted from SK-UT-1-FASN and SK-UT-1-EV cells. Correlative primers are shown in S1 File. Relative quantification method was used to calculate the fold-change of genomic DNA and mRNA expression.

### Proliferation and cell motion assay

2000 SK-UT-1-FASN and SK-UT-1-EV cells were seeded in 96 well plates for 72 hrs. Proliferation was determined by fluorescence-based digital image microscopy (DIMSCAN; Bioimaging Solutions, Inc., San Diego, CA) as previously described [13]. Survivial of siRNA and scrambled control siRNA transfected SK-LMS-1 cells at 72 hr were similarly determined. Cumulative cell motion was measured and calculated with Image-Pro Premier 9.1.4 (Media Cybernetics, Rockville, MD). 5000 SK-UT-1-FASN and SK-UT-1-EV cells were seeded on plastic or fibronectin pre-coated 4 chamber dishes, and individual cells were tracked for movement through 72 hr time-lapse video microscopy.

### Scratch-wound assay

SK-UT-1-FASN and SK-UT-1-EV cells were grown in 6-well plates until 90% confluent. They were then scratched with a 1 ml pipette tip. Selected fields were marked, and microscopic images (magnification 4X) were taken at 0, 24, and 48 hrs post wound introduction. Similarly, SK-LMS-1 cells were transfected with FASN or scramble siRNA at 40% confluency. On the second day following transfection, a wound line was introduced, and microscopic images were taken at the same time points.

### Statistical analysis

Results were presented as the mean ± SD of three independent experiments. The significance of differences was evaluated by paired or unpaired Student's *t* test. A *p* value of less than 0.05 was considered significant.

## Results

### FASN and its principle end-product, palmitate promotes histone 3 modification

**Fig 2:**
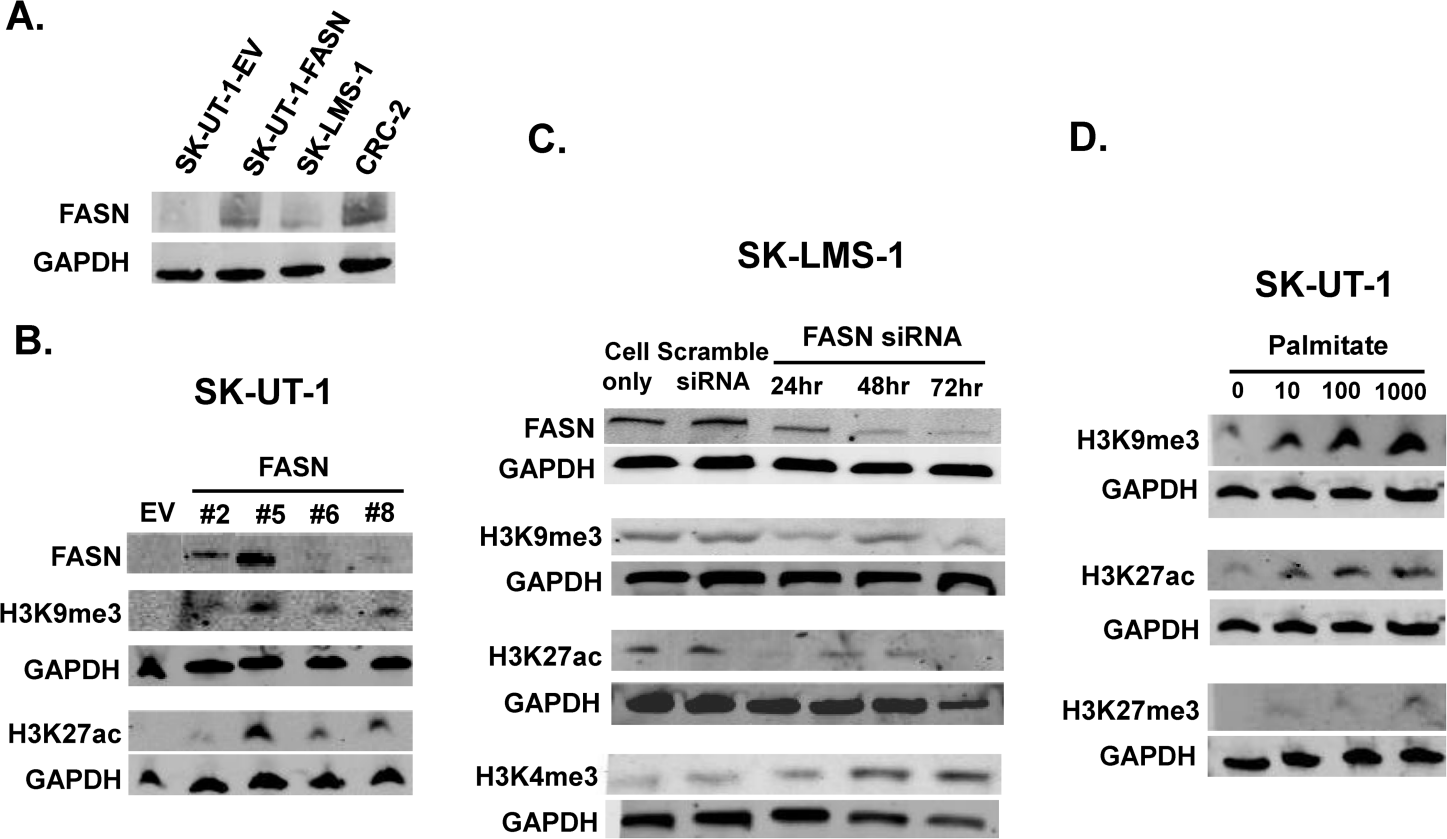
FASN and palmitate modifies H3K methylation and acetylation in Ut-LMS. **(A-B)** Immunoblot showing FASN expression in SK-LMS-1 and FASN- or EV-transduced SK-UT-1 and Ut-LMS CRC cells. FASN was introduced by retroviral vector and positive clones (#2, 5, 6, 8) were selected by puromycin. Clone (#5) with the highest FASN expression was amplified. Non-transformed Ut-LMS cell line CRC-2 was established from patient tissue, and expresses FASN at a high level. **(B)** Time-dependent H3K alterations mediated by forced FASN overexpression in SK-UT-1. **(C)** H3K alterations mediated by FASN siRNA knockdown in SK-LMS-1. Scramble or FASN siRNA were transfected into high FASN expressing SK-LMS-1 cells and lysates were harvested at indicated time points. **(D)** Palmitate reproduces H3K modification in dose dependent manner in low FASN expressing parental SK-UT-1 cells. Cells were treated with palmitate at indicated dose (μM) for 48hr, and cell lysates were harvested for histone 3 detection. The images are representative of at least three experiments. Cropped blots are from gels run under same condition. **FASN**, fatty acid synthase; **EV**, empty vector; **Ut,** uterine; **LMS,** leiomyosarcoma. **CRC**, conditionally reprogrammed cells.

Endogenous FASN expression was detected in SK-UT-1and SK-LMS-1 Ut-LMS cell lines by immunoblotting. As shown in Fig 2A SK-UT-1 has low FASN expression, whereas SK-LMS-1 has high FASN expression. FASN-expressing (-FASN) or empty vector (-EV) human Ut-LMS SK-UT-1 cell lines were produced by transduction with retrovirus prepared from pBabe-FASN-HA-Flag and pBabe-EV-HA-Flag plasmids. FASN-expressing clones were selected in puromycin and clone (#5) with the highest FASN expression was amplified for further studies (Fig 2B).

Histone methylation and acetylation patterns in two common repressive histone 3 (H3) marks, lysine 9 of histone 3 (H3K9me3) and lysine 27 (H3K27me3), and two activating H3 marks, lysine 27 (H3K27ac) and lysine 4 (H3K4me3), were examined in SK-UT-1-FASN cells by immunoblotting. Fig 2B shows that forced expression of FASN promotes trimethylation of H3K9 and acetylation of H3K27 compared to empty vector. FASN overexpression did not significantly alter the trimethylation of H3K4 nor H3K27 (S1A Fig). To confirm these results, Fig 2C shows siRNA targeting of FASN in high FASN-expressing SK-LMS-1 cells inhibits time-dependent trimethylation of H3K9 and acetylation of H3K27. In contrast, trimethylation of H3K4 is increased by FASN knockdown. These results demonstrate that FASN enhances methylation and acetylation of selected H3 lysines that repress and activate transcription in Ut-LMS cell lines.

The predominant FA product of FASN is palmitate. To evaluate FAs as signals for FASN-mediated chromatin remodeling, we examined whether palmitate could reproduce the same H3 modifications as FASN overexpression in low FASN-expressing parental SK-UT-1 cells. Fig 2D shows palmitate induces the same dose-dependent H3K9me3 and H3K27ac modifications as FASN. Further, Fig 2D shows a weak, but dose-dependent increase in H3K27 trimethylation not observed with forced expression of FASN. More interestingly, palmitate overcomes dowregulation of FASN by siRNA in high FASN-expressing partental SK-LMS-1 cells. As shown in S1B Fig, 100 μM palmitate sucessfully rescues FASN expression to a level similar to parental or scrambled siRNA-transfected cells.

### Establishment of primary, non-transformed Ut-LMS cells

Due to the limited availability of Ut-LMS cell lines for study, non-transformed “conditionally reprogrammed cells” (CRCs) have recently been established from patient-derived Ut-LMS in collaboration with Dr. Xuefeng Liu (Georgetown University, Washington, DC) in a City of Hope Institutional Review Board (IRB)-approved protocol. Here, primary Ut-LMS cells are combined with a feeder layer of irradiated murine fibroblasts and a Rho kinase (ROCK) inhibitor [14]. Eventually, the CRCs are transitioned off the feeder layer of fibroblasts, and grow independently. Like pluripotent stem cells, CRCs can grow indefinitely without genetic drift, and are transplantable into immunocompromised mice. Fig 2A shows successful establishment of Ut-LMS CRC-2 cells, which show high FASN expression similar to SK-LMS-1.

### FASN is expressed in LMS clinical samples

FASN expression was detected by immunohistochemistry (IHC) in an 80 core LMS tissue microarray (TMA) containing 30 cases of Ut-LMS. FASN expression was graded 0–3+ by a blinded pathologist. 37/80 (46%) of the LMS revealed 1–3+ expression, and 13/30 (43%) Ut-LMS showed 1–2+ expression. S2A–S2D Fig shows FASN IHC 0–3+ expression in TMA cases. S2E Fig shows a trend toward increased FASN expression in Ut-LMS using American Joint Committee on Cancer (AJCC) grading in this limited clinical sampling.

### FASN alters activity of histone 3-modifying enzymes

**Fig 3:**
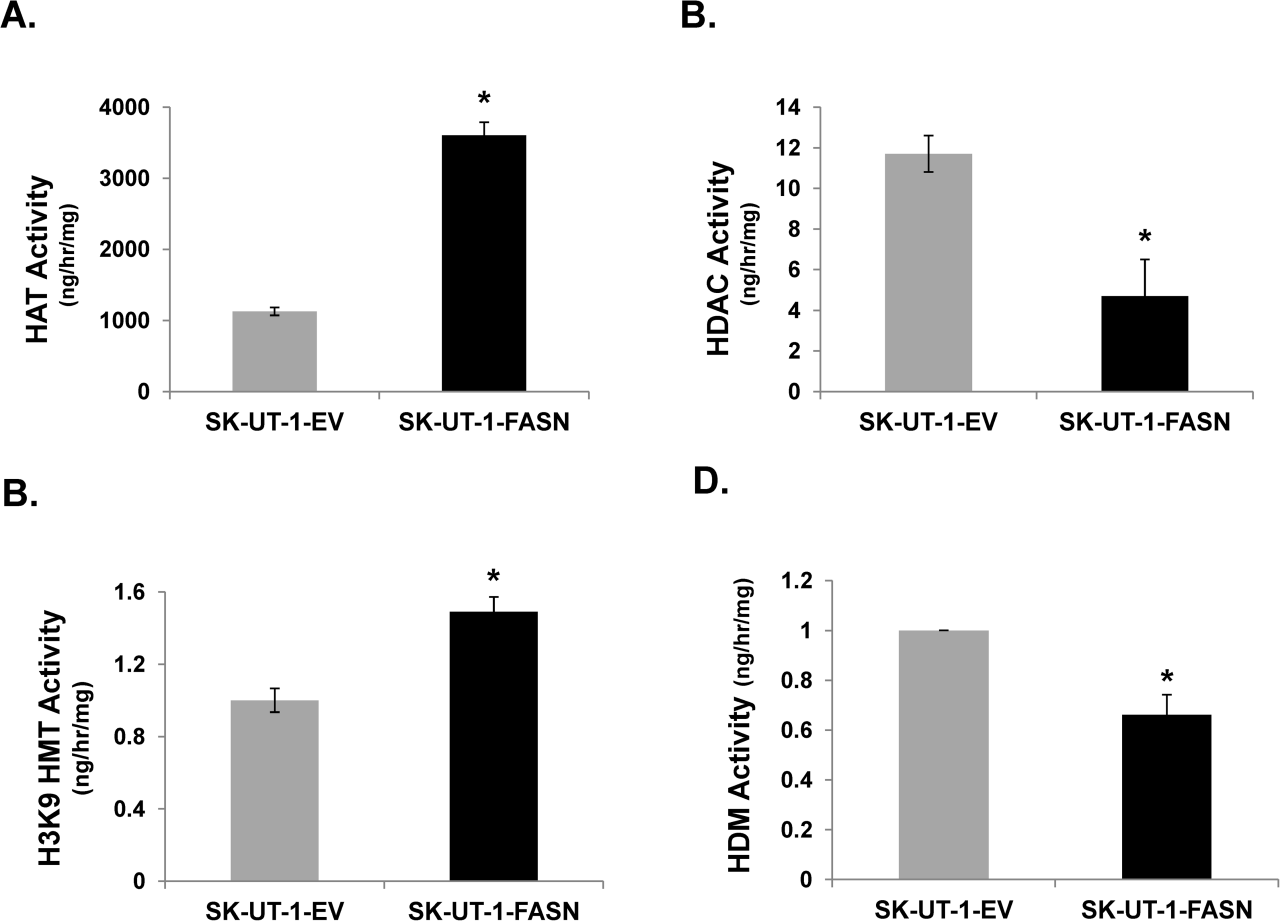
FASN alters H3K9 acetylation and methylation enzyme activities. SK-UT-1-EV or–FASN cells were lysed, and nuclear extract was subjected to ELISA to measure the following histone modification enzyme activities: **(A)** Histone Acetylase (HAT); **(B)** Histone Deacetylase (HDAC); **(C)** Histone Methyltransferase (HMT); **(D)** Histone Demethylase (HDM). *, p<0.05 FASN-transduced SK-UT-1 vs. EV-transduced SK-UT-1 cells.

Histone-modifying enzymatic activities were measured in SK-UT-1-FASN and -EV cells, SK-UT-1, and SK-LMS-1 parental cells. Fig 3A–3D demonstrates that FASN induces histone acetyl transferase (HAT) and methyl transferase (HMT) activities, whereas it inhibits histone deacetylase (HDAC) and demethylase (HDM) activities. S3 Fig confirms high expression of FASN correlated with higher activities of histone methylation and acetylation and reduced activities of histone, demethylation and deacetylation. These results show FASN promotes histone 3 methylation and acetylation through alteration of histone modifying enzymatic activities.

### FASN-mediated enhances H3K9me3 genomic binding and alters gene expression

**Fig 4:**
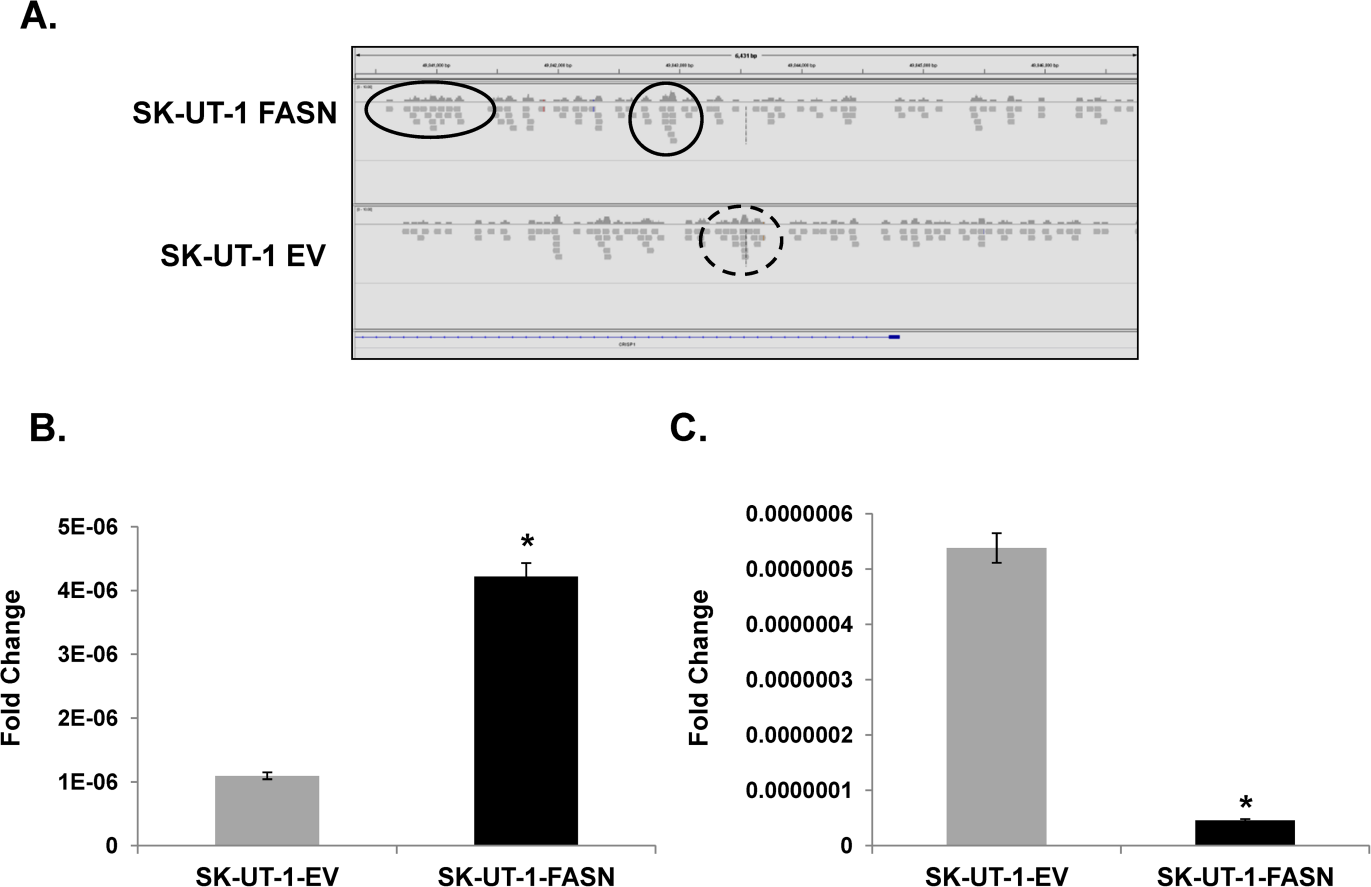
FASN induces H3K9me3 to repress CRISP1 expression. (**A**) ChIP-seq was performed in SK-UT-1-EV and–FASN cells with anti-H3K9me3 antibody. Enriched genomic regions were compared between SK-UT-1-FASN and SK-UT-1-EV. Screenshot shows *CRISP1* is enriched for H3K9me3 in SK-UT-1-FASN cells (solid line) vs. SK-UT-1-EV (dot line); **(B)** Enrichment of *CRISP1* binding to SK-UT-1-FASN genomic DNA by ChIP-PCR. Immunoprecipited chromatin DNA by H3K9me3 antibody was reversed crosslinking, purified and subject to q-PCR for *CRISP1* gene. **(C)** qRT-PCR for *CRISP1* expression. SK-UT-1-EV or–FASN cells were lysed for total RNA extraction, cDNA synthesis and q-PCR of *CRISP1*. *, p<0.05 FASN-transduced SK-UT-1 vs. EV-transduced SK-UT-1 cells. Data was normalized to GAPDH or β-actin. Results are expressed as the percentage of input DNA.

Chromatin immunoprecipitation with massively parallel DNA sequencing (ChIP-seq) in SK-UT-1-FASN and SK-UT-1-EV cell lines was performed with anti-H3K9me3 antibody to identify genomic regions differentially-enriched for H3K9me3 binding. 289 genomic regions were enriched ≥ 2-fold, and 329 regions were reduced ≥ 2-fold in SK-UT-1-FASN compared to SK-UT-1-EV cells in genome-wide analyses (sequence data are available at https://www.ncbi.nlm.nih.gov/geo/query/acc.cgi?token=yzcheumixbchpmn&acc=GSE98557). We focused on the greatest differentially-regulated exonic and intergenic regions enriched in proximal and distal regulatory regions (-8 to +2 kb) of adjoining genes (Integrative Genomics Viewer, Broad Institute, Boston, MA) [15]. We chose to investigate further one of the differentially-enriched genes, *CRISP1*. *CRISP1* is primarily expressed in male, and to lesser extent in female reproductive tracts, where it functions to modulate fertilization [16]. Fig 4A shows FASN induces H3K9me3 binding to the *CRISP1* promoter, and confirmed by quantitative polymerase chain reaction (qPCR) (Fig 4B). Consistent with its transcriptionally repressive activity, enhanced H3K9me3 binding results in reduced *CRISP1* expression (Fig 4C). Further, S4 Fig suggests that repression of *CRISP1* is related to the lipogenic phenotype because treatment of parental SK-UT-1 cells with palmitate recapitulates *CRISP1* repression.

### Forced expression of FASN induces proliferation of Ut-LMS cell lines

**Fig 5:**
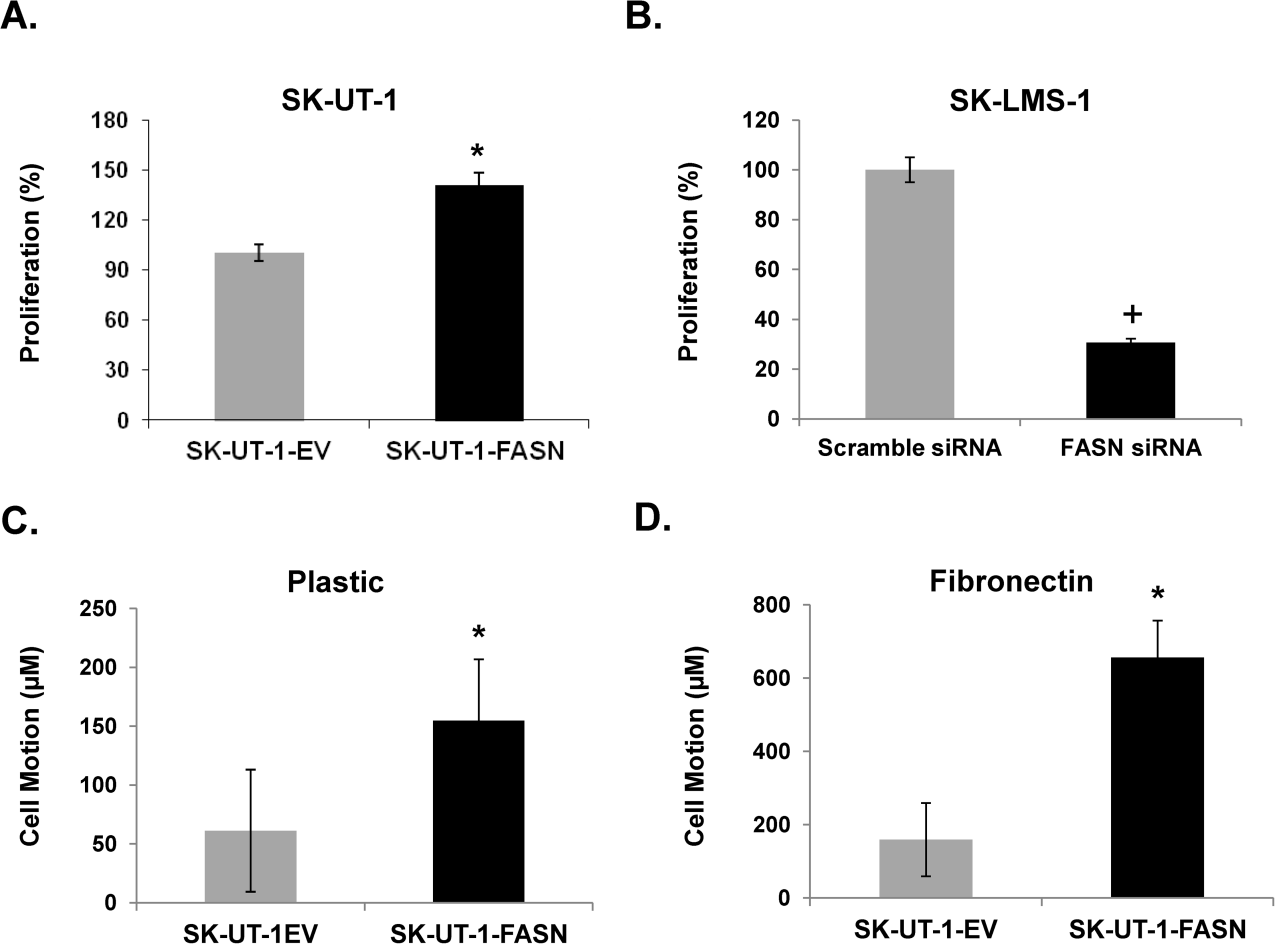
FASN promotes Ut-LMS proliferation and increases Ut-LMS motion. (**A)** DIMSCAN assay in SK-UT-1-EV and -FASN cells after 72 hrs incubation in 96 wells plate; (**B**) DIMSCAN assay in FASN siRNA-transfected SK-LMS-1 cells at 72 hr. **Quantified cell motion detected by 72 hr time-lapse video microscopy.** (**C**)Plastic plate; (**D**)Fibronectin-coated plate. Cell motion was recorded, tracked and quantitated by Image-Pro Premier 9.1.4. *, p<0.05 FASN-transduced SK-UT-1 vs. EV-transduced SK-UT-1 cells; +, p<0.05 FASN siRNA transfected SK-LMS-1 vs. scramble siRN transfected SK-LMS-1 cells.

Fig 5A shows FASN overexpression SK-UT-1 cells increases proliferation 41% over EV as determined by fluorescence-based digital image microscopy (DIMSCAN). In contrast, Fig 5B shows siRNA-mediated knockdown of FASN reduces SK-LMS-1 cell survival by nearly 70% compared with cells transfected with scramble siRNA.

### FASN enhances motion and migration in Ut-LMS cell lines

**Fig 6:**
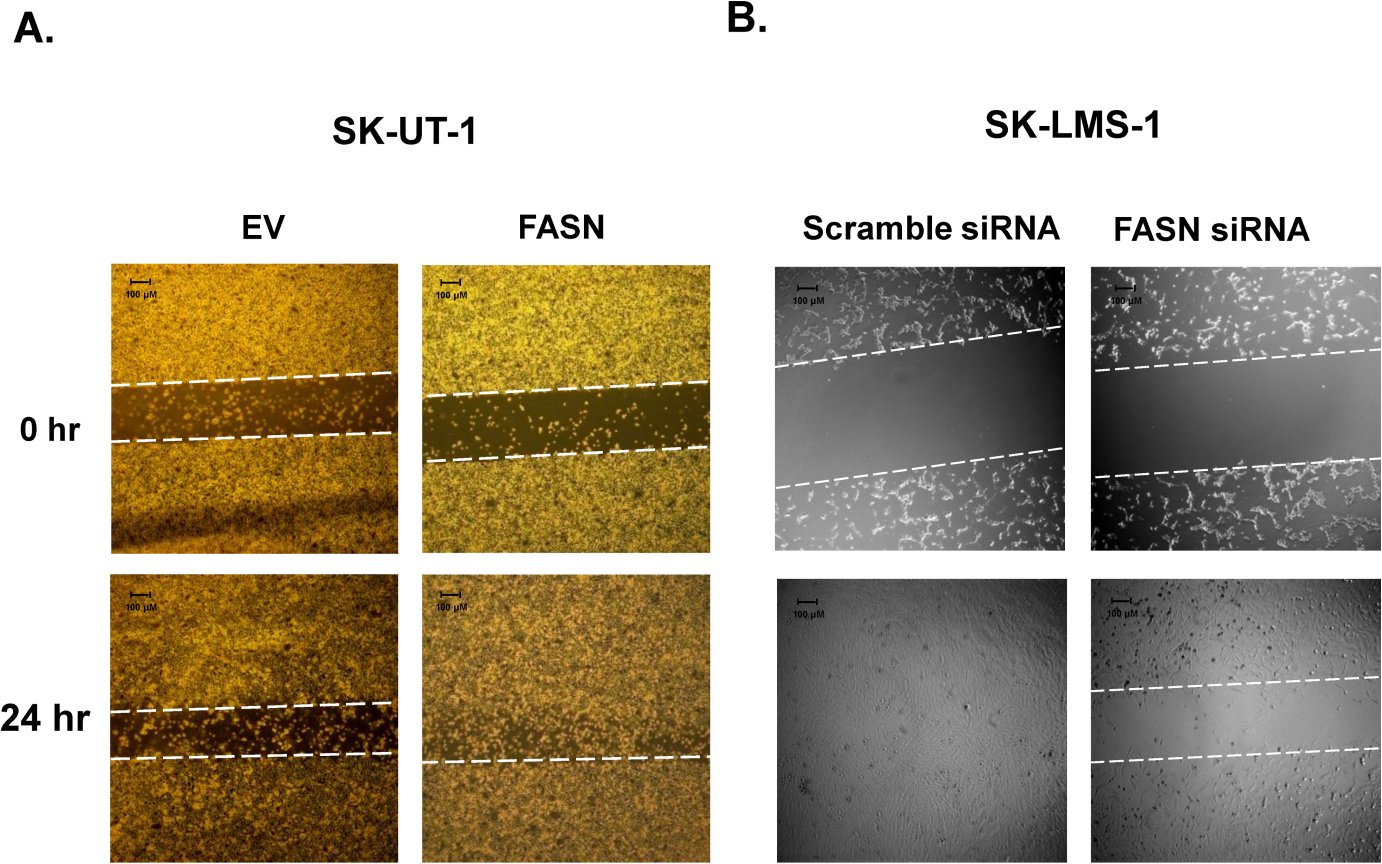
Scratch-wound assay of Ut-LMS. (**A**) Migration of SK-UT-1-EV and SK-UT-1-FASN cells at 0 and 24 hr after introducing “wound.” (**B**) Migration of SK-LMS-1 cells at 24 (0 hr) and 48 (24 hr) hr after transfection with scrambled siRNA or siRNA targeting FASN (wound introduction).

Cellular motion was quantified by time-lapse microscopy on plastic and fibronectin-coated plates, and migration of Ut-LMS cells was examined by scratch-wound assay. Fig 5C and 5D shows forced expression of FASN increases cell motility in SK-UT-1 cells by 153% in plastic and 313% in fibronectin over the 72 hr time period measured. Fig 6A demonstrates that forced expression of FASN in SK-UT-1 cells enhances migration at 24 hr after introducing a scratch wound in the tissue well. In contrast, Fig 6B shows siRNA-mediated knockdown of FASN inhibits SK-LMS-1 cell migration, whereas there is a complete “closure of the wound” in cells transfected with scrambled FASN siRNA.

## Discussion

FASN is an attractive therapeutic target. FASN inhibitor-mediated cytotoxicity results from accumulation of toxic intermediary metabolite malonyl-CoA, induction of endoplasmic reticulum (ER) stress, accumulation of ceramide, and caspase activation [17–20]. A phase I clinical trial of a first-in-class FASN inhibitor, TVB-2640, in patients with solid tumors (ClinicalTrials.gov identifier NCT02223247) shows preliminary anti-tumor activity [21]. Further, a recent study shows inhibition of ACC suppresses fatty acid synthesis and tumor growth of non-small-cell lung cancer in preclinical models [22].

Both *de novo* lipogenesis and epigenetic reprogramming promote the sarcoma phenotype [6, 23–26]. The EWSR1-FLI-1 fusion protein pathognomonic for Ewing sarcoma (ES) binds the promoter of histone methyltransferase (HMT) enhancer of Zeste, *Drosophila*, Homolog 2 (EZH2) to promote expression in ES [23]. EZH2-mediated gene silencing is dependent upon histone deacetylase (HDAC) activity, and down-regulation of EZH2 by RNA interference (RNAi) suppresses ES oncogenic transformation [23]. The SYT-SSX2 fusion protein present in one-third of synovial sarcomas (SS) interacts with components of the Polycomb repressive and SWI/SNF chromatin-remodeling complexes, which leads to global alteration of nuclear programming and tumor initiation [24]. Dedifferentiated liposarcomas (DLPS) harbor histone deacetylase 1 (*HDAC1*) mutations in 8.3% of cases [25], and liposarcoma methylomes show alteration in differentiation genes, including CCAAT/Enhancer Binding Protein Alpha (*CEBPA*) methylation in 24% of cases. Treatment with demethylating agents restores *CEBPA* expression in DLPS, is antiproliferative and proapoptotic *in vitro* and reduces tumor growth *in vivo*. Further, DLPS show elevated H3K9me3, and integrated chromatin immunoprecipitation-sequencing (ChIP-seq) with gene expression analyses show H3K9me3 mediates differential regulation of genes involved in cellular differentiation and migration through inhibition of Kruppel-like factor 6 (*KLF6*) [26]. These reports highlight the role of altered histone modification and DNA methylation in the pathobiology of bone and STS.

We report here that FASN enhances the malignant phenotype in Ut-LMS as demonstrated by increased proliferation, migration, and cellular motion. FASN promotes selected H3 trimethylation and acetylation through alteration of H3-modifying enzymatic activities. Palmitate, the principle end-product of FASN, recapitulates the FASN-mediated H3 modifications in a dose-dependent fashion, which links palmitate to FASN-mediated epigenetic signaling. Finally, ChIP-seq targeting H3K9me3 identified multiple regions of enriched binding, which we have shown to be functionally important as demonstrated by the transcriptional repression of *CRISP1*. These results strikingly implicate the mechanistic pathway through which the “lipogenic phenotype of cancer” mediates its cellular effect is through altered chromatin remodeling.

## Supporting information

S1 Fig **(A)** FASN overexpression does not alter H3K4me3 and H3K27me3 in SK-UT-1 cells. **(B)** Palmitate rescues FASN expression in SK-LMS-1 cells transfected with siRNA targeting FASN.
(DOCX)

S2 Fig FASN is expressed in human LMS and increases with AJCC tumor grade. (DOCX)

S3 Fig FASN alters H3K9 acetylation and methylation enzyme activities. (DOCX)

S4 Fig Palmitate reproduces CRISP1 repression. (DOCX)

S5 Fig Original blots. (DOCX)

S1 File Primers for ChIP-PCR and RT-PCR. (DOCX)
