# Supplementary material for: Fatty acid synthase reprograms the epigenome in uterine leiomyosarcomas
Source: PLoS One. 2017 Jun 27;12(6):e0179692. doi: 10.1371/journal.pone.0179692 (PMC5487038; doi:10.1371/journal.pone.0179692)
Supplement: S1 Fig — (A) FASN overexpression does not alter H3K4me3 and H3K27me3 in SK-UT-1 cells. (B) Palmitate rescues FASN expression in SK-LMS-1 cells transfected with siRNA targeting FASN. (DOCX) [file pone.0179692.s001.docx]

**S1 Fig. (A) FASN overexpression does not alter H3K4me3 and H3K27me3 in SK-UT-1 cells. (B) Palmitate rescues FASN expression in SK-LMS-1 cells transfected with siRNA targeting FASN.** Immunoblot showing FASN expression in high FASN-expressing SK-LMS-1 cells and SK-LMS-1 cells transfected with scramble or FASN-targeted siRNA. Lysates were harvested at the indicated time points (lane 1-4). 24 hr after FASN siRNA tranfection, the cells were treated with palmitate at indicated dose (µM) for an additional 48hr, and the cell lysates were harvested for FASN detection (lane 5-6).

**B**

**A**
