# Supplementary material for: Fatty acid synthase reprograms the epigenome in uterine leiomyosarcomas
Source: PLoS One. 2017 Jun 27;12(6):e0179692. doi: 10.1371/journal.pone.0179692 (PMC5487038; doi:10.1371/journal.pone.0179692)
Supplement: S2 Fig — (DOCX) [file pone.0179692.s002.docx]

**S2 Fig. FASN is expressed in human LMS and increases with AJCC tumor grade.** FASN expression was detected by immunohistochemistry (IHC) in an 80 core LMS tissue microarray (TMA) containing 30 cases of Ut-LMS. FASN expression was graded 0-3+ by a blinded pathologist. 37/80 (46%) of the LMS revealed 1-3+ expression, and 13/30 (43%). **(A)** Ut-LMS, 0+; **(B)** Ut-LMS, 1+; **(C)** Ut-LMS, 2+; **(D)** Smooth muscle LMS, 3+. **(E)**Total number of Ut-LMS cases and cases with 1-3+ IHC FASN expression. **AJCC,** American Joint Committee on Cancer.
