# Supplementary material for: Fatty acid synthase reprograms the epigenome in uterine leiomyosarcomas
Source: PLoS One. 2017 Jun 27;12(6):e0179692. doi: 10.1371/journal.pone.0179692 (PMC5487038; doi:10.1371/journal.pone.0179692)
Supplement: S3 Fig — (DOCX) [file pone.0179692.s003.docx]

**S3 Fig. FASN alters H3K9 acetylation and methylation enzyme activities.** Low FASN-expressing SK-UT-1 or high FASN-expressing SK-LMS-1 cells were lysed, and nuclear extract was subjected to ELISA to measure the following histone modification enzyme activities: **(A)** Histone Acetylase (HAT); **(B)** Histone Deacetylase (HDAC); **(C)** Histone Methyltransferase (HMT); **(D)** Histone Demethylase (HDM). *, p<0.05, SK-UT-1 vs SK-LMS-1 cells.
