# Supplementary material for: Fatty acid synthase reprograms the epigenome in uterine leiomyosarcomas
Source: PLoS One. 2017 Jun 27;12(6):e0179692. doi: 10.1371/journal.pone.0179692 (PMC5487038; doi:10.1371/journal.pone.0179692)
Supplement: S4 Fig — (DOCX) [file pone.0179692.s004.docx]

**S4 Fig. Palmitate reproduces *CRISP1* repression.** qRT-PCR for *CRISP1* expression. SK-UT-1 parental cells treated with palmitate at indicated dose (µM) for 48 hr were lysed for total RNA extraction, cDNA synthesis and q-PCR of *CRISP1*. *, p<0.05 palmitate treated SK-UT-1 vs. control treated SK-UT-1 cells. Data are expressed as the percentage of input DNA.
