# Supplementary material for: Fatty acid synthase reprograms the epigenome in uterine leiomyosarcomas
Source: PLoS One. 2017 Jun 27;12(6):e0179692. doi: 10.1371/journal.pone.0179692 (PMC5487038; doi:10.1371/journal.pone.0179692)
Supplement: S5 Fig — (DOCX) [file pone.0179692.s005.docx]

**S5 Fig. Original blots. (A)** gel for Fig. 2A. **(B)** gels for Fig. 2B. **(C)** gels for Fig. 2C. **(D)** gels for Fig. 2D. **(E)** gel for S1 Fig.
