# Supplementary material for: Fatty acid synthase reprograms the epigenome in uterine leiomyosarcomas
Source: PLoS One. 2017 Jun 27;12(6):e0179692. doi: 10.1371/journal.pone.0179692 (PMC5487038; doi:10.1371/journal.pone.0179692)
Supplement: S1 File — (DOCX) [file pone.0179692.s006.docx]

**S1 File. Primers for ChIP-PCR and RT-PCR.** *CRISP1* DNA 5’ primer tcaaaccccaaactgcaaa; 3’ primer ttttgttcgctacccattca; *CRISP1* RNA 5’ primer ttgggccacatcttacctga; 3’ primer gttacttgggcaggcttcac.
